# Holacracy, a modern form of organizational governance predictors for person-organization-fit and job satisfaction

**DOI:** 10.3389/fpsyg.2022.1021545

**Published:** 2023-01-19

**Authors:** Lena Weirauch, Sibylle Galliker, Achim Elfering

**Affiliations:** Department of Psychology, University of Bern Fabrikstrasse, Bern, Switzerland

**Keywords:** person–organisation fit, holacracy, self-organisation, big five, job satisfaction

## Abstract

This study compares illegitimate tasks and appreciation in traditional work organisations and holacracy work organisations based in Switzerland and Germany. In addition, the study tests whether the fit between employees and holacracy organisations depends on personality characteristics. Ninety-five employees working in holacratic companies participated in an online survey with standardised questionnaires on illegitimate tasks, Big Five personality dimensions, perceived holacracy satisfaction and person–organisation fit. For the comparison of illegitimate tasks and appreciation, a propensity-matching comparison group of people working in traditional companies was used. The results revealed significantly lower illegitimate tasks t(53) = −2.04, *p* < 0.05, with a lower level (2.49) in holacracy than in traditional work (2.78). Concerning appreciation, the results showed significantly higher values for holacratic (5.33) than for traditional work [4.14, t(53) = 4.86, *p* < 0.001]. Multiple linear regression of holacracy satisfaction on personality dimensions showed neuroticism (b = −4.72, *p* = 0.006) as a significant predictor. Agreeableness showed marginally significant results (b = 2.39, *p* = 0.06). This indicates that people scoring low on neuroticism and high in agreeableness may thrive better in holacracy organisations. Based on the results, theoretical and practical implications as for example implications for corporates hiring strategy, are discussed. Finally, this study presents numerous directions for future research.

## 1. Introduction

Holacracy is currently defined as a comprehensively designed internal management system; it has received significant attention for its adoption at Zappos and other big companies (Bernstein et al., 2016), especially the fast-growing sector of technology and start-ups practice holacracy (Kaduthanam and Heim, 2019). Currently, holacracy is the most widely adopted system of organisational self-management (Bernstein et al., 2016). According to the official website of holacracy, more than 1,000 companies all over the world are using the holacracy approach (Who’s Practicing?, n.d.). Although holacracy is becoming more popular, research on working conditions and well-being of employees working in holacratic organisations is sparse.

The concept of holocracy refers to the development of a new way of structuring and running an organisation that replaces conventional management. That was the goal of the entrepreneur Brian Robertson (Groth, 2014). When Robertson founded his own tech company, he realised that companies nowadays are confronted with a world that is changing more quickly than ever before and that working today means something completely different from before 30 years (Hackl et al., 2017). In his view, the existing methods did not fulfil his demands and traditional forms of organisation would not allow him to react to changes fast enough, and therefore it avoids a successful product development. Consequently, based on the principle of trial and error, his goal was to create a method that allows companies to work together in a more effective and powerful way (Robertson, 2007). In holacratic companies, authority and decisions are distributed throughout circles, and employees no longer have titles anymore but have roles. Roles include a clear objective regarding how they contribute to the organisation and their circle. Once the role no longer provides a benefit, it is removed. Individuals can also hold multiple roles, such as “lead-link,” “rep-link,” “facilitator” or “secretary.” The lead-link represents the circle during meetings, the rep-link represents sub-circles (Van De Kamp, 2014), the facilitator leads the circle through the given holacratic process and the secretary maintains circle records and schedules meetings (Robertson, 2007). Another essential characteristic of holacracy is the constitution of governance guides, which does not specify the implementation of the tasks but how circles must be established and operated. The constitution is stipulated according to which guidelines new roles should arise and how circles should communicate among one another (Bernstein et al., 2016). Again, there is a gap between expectations on how holacracy change work tasks and the empirical evidence on change in work tasks.

In summary, development of holacratic organisation was triggered by task needs but not based on work design theory. Nevertheless, holacratic organisation can be expected to change not only the organisational structure but also the work tasks. To get a better understanding of how holacracy affects employees, the study aims to find out whether specific work conditions like unnecessary tasks and unreasonable work tasks are less common in holacratic work organisation than in traditional work. Moreover, work appreciation is expected to be higher in holacratic compared to traditional work organisation. Therefore, a holacratic work organisation company and a comparison group consisting of people working in traditional companies was examined. Current studies underline the need to systematically analyse new forms of work organisation using work psychology tools and methods (Junker et al., 2022). The first part of this study focuses on differences in work conditions between holacratic and traditional organisations due to increasing relevance and interest in new forms of organisations (Csar, 2017). The goal is to understand and evaluate better these new forms of organising companies by comparing important work conditions, not just organisational structures, at workplaces. The second part of the study focuses on different personality traits as predictors for Person‐organisation fit (PoF) and satisfaction with holacracy. The objective is to identify relevant personality traits that predict satisfaction and individual fit with holacracy. So far, only little is known regarding predictors of a good fit for employees within this new organisational form. Some papers used expert interviews (Van De Kamp, 2014), but to the best of our knowledge, no study has explicitly examined predictors of the person–organisation fit in companies implementing holacracy.

### 1.1. Unnecessary and unreasonable tasks in a holacratic work organisation

In a rapidly changing work environment, deficits in job design are a frequent reason why employees sometimes must complete tasks that are perceived as unnecessary or unreasonable because the tasks do not fit to their occupational role. Unnecessary and unreasonable tasks are described by a psychological construct called illegitimate tasks (Semmer et al., 2010, 2015; Kottwitz et al., 2019). These tasks occur when employees are faced with tasks they feel do not match their occupational role. Semmer et al. (2015) found that illegitimate tasks predicted strain and should, as a result, be seen as a part of job design. This study seeks to investigate whether people who work in holacratic companies experience fewer illegitimate tasks through the concept of self-organisation and high autonomy. One of the main characteristics of holacracy is the “self-governing” approach. Robertson himself explains holacracy as a “rule system for anarchy,” thus a system without rulers (Groth, 2014). This results in the selection of tasks and an approach to complete them which is left open to individual discretion (Robertson, 2015). Moreover, in holacratic companies, people work with roles that are constantly being changed or even redefined (Van De Kamp, 2014). This should result in employees experiencing illegitimate tasks less often, as tasks that are perceived to be illegitimate can either be quickly relinquished by adjusting roles, and thus feeling that the new task fits the different roles after all, or by someone else taking over the task and assuming it. According to Semmer et al. (2015), “illegitimate tasks send an implicit message of disrespect that represents a potential threat to the self.” The results are noteworthy, because a recently published meta‐analysis highlighted that self‐esteem had significant prospective effects on job satisfaction, job success, and job resources (Krauss and Orth, 2022). In the case that holacratic structures lead to fewer illegitimate tasks, this would result in a meaningful outcome for organisational psychology.

*Hypothesis 1*. People who work in holacratic companies will experience fewer illegitimate tasks than people who work in traditional companies.

### 1.2. Appreciation at work in holacracy

Appreciation at work is an important resource that matches illegitimate tasks as a resource when dealing with different roles (Semmer et al., 2015). Illegitimate tasks are often perceived as implying a lack of appreciation and respect. The idea behind this is that the person to whom the task has been assigned concludes that the person assigning the task has not dealt with their own interests (Kottwitz et al., 2019). Results from Stocker et al. (2010) also showed that (lack of) appreciation mediated the effect of illegitimate tasks on job satisfaction. The importance of appreciation in the workplace is shown by the results of a study by Siegrist (1996), who identified appreciation as an important reward factor and as having a direct influence on job satisfaction (Elfering et al., 2007). In addition to its resource value regarding illegitimate tasks at work, appreciation is a powerful general resource provided by others (Elfering et al., 2007). Specifically, perceived appreciation means being recognised as a valuable person. As a result, self-esteem is strengthened, leading to well-being and higher job satisfaction (Elfering et al., 2007). Appreciation is often associated with leadership and appreciation from the supervisor (Kottwitz et al., 2019). In holacratic companies, supervisors and managers do not exist (Robertson, 2015), but the team has a very high priority. The concept’s aim is to create an environment where everyone contributes equally to the success of the company due to missing hierarchies, and everyone follows the same purpose (Robertson, 2007). Moreover, in holacracy there is more and quicker feedback on work processes from more feedback providers. Consequently, employees receive more positive feedback, which is a primary source of work appreciation (Semmer et al., 2015). The absence of hierarchy and improved feedback contribute to a respectful work climate and appreciation among the team (Kottwitz et al., 2019). Results of a recently published study support these findings, which identified that holacracy creates an opportunity for appreciation since all tasks and functions become visible and cannot be overlooked anymore (Schell and Bischof, 2021). Moreover, the fact that in meetings roles and tasks are constantly reviewed leads to high transparency and awareness regarding the importance of each task (Schell and Bischof, 2021). Based on these findings, the following hypothesis was formulated:

*Hypothesis 2*. People who work in holacratic companies will experience higher appreciation than people who work in traditional companies.

### 1.3. Person–organisation fit in holacracy

A second research question refers to personality as a predictor for person–organisation fit in holacracy. One of the most known implementations of holacracy is Zappos, an online retailer with 1,500 employees in 2014 (Van De Kamp, 2014). When the CEO of Zappos decided to introduce holacracy, he sent a letter to his employees stating that self-management and self-organisation would become the most important requirements. But “self-management and self-organisation is not for everyone, and not everyone will want to move forward in the direction,” he said. That is why he offered severance packages to the employees for whom holacracy was not a good fit at their own discretion (Bernstein et al., 2016). Researchers also claim that holacracy will most likely not work for everyone (Van De Kamp, 2014; Schermuly, 2019). Van De Kamp (2014) states that people who have insufficient self-management skills might face difficulties with holacracy. Rough estimates that a potential lack of fit with holacracy can be inferred from numbers reported at Zappos, i.e., the 18% of Zappos employees who decided to take the severance package and leave the company (Bernstein et al., 2016). A recent study argues that in many cases new work approaches are focusing on the structures of companies instead of on the people who work there. According to Schermuly (2019), most of the new work initiatives fail because of that. Depending on personality characteristics, the same structures at work can be perceived differently. Thereby, the present study aspires to investigate for whom holacracy will probably be a good person-organisation fit. Person–organisation fit is the compatibility between employees and an organisation (Kristof, 1996). Identifying predictors may be important for human resources managers, who in the future could primarily employ those who are likely to be satisfied with holacratic structures and might benefit from the various advantages of person–organisation fit. Companies could also use these predictors to avoid employee resignations due to a low person–organisation fit (Kristof-Brown et al., 2005). In order to capture a comprehensive taxonomy of personality through a manageable number of items, this study uses the five-factor model (Ostendorf, 1990; Costa and McCrae, 1992). Studies show that personality is also associated with self-regulation, as it generates individual differences in emotion, thought and behaviour (Gramzow et al., 2004). Johnson et al. (2013) showed that self-regulation is closely linked to the person–environment fit and claim, and that person–environment fit should be seen through the lens of a self-regulation framework. They conceptualised person–environment fit as a discrepancy between the ideal conditions for people and the experienced conditions.

Accordingly, the assumption of this study is that holacracy, with all its facets, requires self-regulatory characteristics that are bound to personality and decide the fit between employee and organisation. The goal is to find what kind of personality fits the holacracy approach and leads to satisfaction with this model, as there is no hierarchy in holacracy anymore.

The five-factor model of personality comprises extraversion, neuroticism, openness to experience, conscientiousness and agreeableness. Each category represents a broad domain that consists of more explicit personality traits (Zhao and Seibert, 2006). A person who scores high on extraversion can be described as active, dominant and enthusiastic (Costa and McCrae, 1992). Low values for extraversion imply that a person prefers to spend time alone and could be described as quiet and reserved. As there is no hierarchy anymore, the distribution of tasks is left open to employees. If someone is more extraverted, this person might get easier access to tasks they would like to accomplish because they are sociable, active and talkative. Also, for people working in a holacratic company, they will be most likely to spend more time with other people because of the constant interactions with their colleagues; ergo, teamwork is key. Thereby this person would feel that the organisation is fulfilling their needs. Taking that into account, it is possible to infer the following:

*Hypothesis 3*. In holacracy, people who score higher on extraversion also score higher on the *person–organisation fit* scale, meaning that a positive correlation between *extraversion* and *person–organisation fit* is expected.

Neuroticism indicates the emotional stability of a person. People with high values for neuroticism could be described as anxious, impulsive and vulnerable. On the other hand, low levels on the scale result in characteristics like calmness, self-confidence and relaxation (Costa and McCrae, 1992). Employees working in holacratic companies experience an unstructured environment that requires a lot of responsibility. Self-confidence and evenness seem to be important characteristics to handle holacratic structures and experience a high *person–organisation fit*. For this reason, the following can be posited:

*Hypothesis 4*. In holacracy, people who score low on *neuroticism* score higher on the *person–organisation fit* scale, meaning that a negative correlation between *neuroticism* and *person–organisation fit* is expected.

Openness to experience covers the desire to constantly discover new things and have new experiences. High values for this dimension imply that a person is innovative, imaginative and interested in art and music, for example. Moreover, it means that they are attentive to the emotions of other people and to their own. Low values in contrast imply that someone prefers routines, is traditional and uncreative (Costa and McCrae, 1992). Some studies have discovered that creativity, which is part of openness to experience, is a predictor for entrepreneurship (Shane and Nicolaou, 2015). For instance, Shane and Nicolaou (2015) found high correlations between creativity and the likelihood of starting a business. In their study, Hamidi et al. (2008) also found high correlations between inventiveness and entrepreneurial intentions. Holacracy, along all its facets, encourages employees to act like an entrepreneur. Additionally, in holacratic companies everyone can contribute their ideas, so there is plenty of room for openness to experience (Krasulja et al., 2016). Considering these different insights, the following hypothesis has been formulated:

*Hypothesis 5*. In holacracy, people who score higher on *openness to experience* score higher on the *person–organisation fit* scale, meaning that a positive correlation between *openness to experience* and *person–organisation fit* is expected.

Conscientiousness describes how organised, hardworking, motivated and self-controlled a person can be. This means that they will be able to formulate goals, to plan them, and to work hard to accomplish them (Costa and McCrae, 1992). In holacracy, self-organisation becomes one of the most important skills. Employees have different roles and responsibilities, so prioritising becomes key. Lack of self-organisation competence could result in overtaxation and could therefore lead to a low *person–organisation fit* because it feels like the employer does not match the required abilities. Therefore, the expectation is as follows:

*Hypothesis 6*. In holacracy, people who score higher on conscientiousness score higher on the *person–organisation fit* scale, meaning that a positive correlation between *conscientiousness* and *person–organisation fit* is expected.

A person with high values for agreeableness tends to be warm, trusting, altruistic and caring. Low values, in contrast, result in ruthlessness, manipulation and mistrust (Costa and McCrae, 1992). According to Bernstein et al. (2016), the time spent in meetings increases in holacratic companies, so a significant number of interactions with other people is always demanded. In order to avoid conflicts and to maintain friendly relationships, employees must show trust, courtesy and cooperativeness, elements that belong to the concept of agreeableness. Individuals who score high on agreeableness should be more eager to interact with others in a positive way without triggering conflicts. Also, they should experience that selfish behaviour is not accepted. Gary Hamel, professor at the London Business School, argues that people who show agreeable behavior will handle holacracy better than egoistic people (Hamel, 2012). A possible explanation for that could come from a recently published study from Hughes et al. (2022) where it was assumed that agreeableness is a relevant factor for determining the extent to which an interpersonal stressor is assessed as a threat to the self-esteem, because agreeable employees are probably more likely to forgive rude or unhelpful behavior and may not perceive this kind of behavior as a threat to self-esteem. Moreover, low values on agreeableness could lead to more dysfunctional support (Semmer et al., 2006) within the team and thereby negatively affect the perceived PoF. Taking this into account, this study will investigate this dimension in more detail because agreeableness is assumed to be a relevant factor for holacracy.

*Hypothesis 7a*. In holacracy, people who score higher on *agreeableness* score higher on the *person–organisation fit* scale, meaning that a positive correlation between *agreeableness* and *person–organisation fit* is expected.

*Hypothesis 7b*. In holacracy, people who score higher on *agreeableness* score higher on the *holacracy satisfaction* scale, meaning that a positive correlation between *agreeableness* and *holacracy satisfaction* is expected.

## 2. Method and materials

### 2.1. Participants

The recruitment of participants for this questionnaire study took place in December 2020 *via* social media, especially *via* LinkedIn, because the social network allowed to contact people without the need for a personal connection. An *a priori* power calculations were performed and a sample size of 100 participants (*N* = 100) was shown. Due to certain exclusion criteria, only 95 responses could be included in the calculations. A total of 240 people clicked on the link, of whom 115 participated in the survey, 95 of whom completed the questionnaire. This resulted in a final sample size of 95 (*N* = 95) participants, 41 of whom were female (*n* = 41) and 44 of whom were male (*n* = 54). The average age of participants was between 31 and 40 years old. The youngest participants were between 20 and 30 years old, and nobody was older than 60 years. Of all participants, 76% were between 20 and 40 years old, 43% were Swiss (the German speaking part), and 57% were German. Due to data protection, the exact age of the respondents was not recorded, as only the age range was requested as a means to avoid drawing conclusions about a person based on their age. Around one third of participants were single, while one third indicated they were in a partnership, and one third were married. All participants were working for a company that used the holacracy approach at the time of the survey. Forty-seven percent of participants worked for a company in the technology sector. In terms of elected roles within holacracy (lead-link, rep-link, facilitator, secretary), most participants (38%) reported having taken the role of lead-link; 13% the role of rep-link and 26% no elected role. All data were collected completely anonymously. The respondents were informed about the content of the study and their voluntary participation. The participants in the comparison sample were employed persons from German-, French- and Italian-speaking Switzerland. In total, 2,846 (*N* = 2,846) participants took part in the study, of whom 46.9% were female and 53.1% male. Eleven percent were between 16 and 24 years old, 34% between 25 and 39 years old, 35% between 40 and 54 years and 18% between the 55 and 65. Most of them spoke German (70%) and worked full-time (Galliker et al., 2020). The language of the study was German, and this evaluation was approved by the Ethics Committee of the University of Bern, Switzerland (Ethics No. 2019–01-00005).

### 2.2. Materials

#### 2.2.1. Questionnaires

The first research question compares a holacratic work organisation to a traditional work organisation with respect to illegitimate tasks and *appreciation*. The second research question uses only the participants who worked in a holacratic work organisation. In this study, *the Big Five* personality dimensions were tested to predict holacracy satisfaction and person–organisation fit. All inquiries, apart from demographic questions and the item on *holacracy satisfaction*, are based on existing questionnaires and scales with good psychometric reliabilities and validities. The demographic section consists of five questions. The first one addresses gender, offering the options “male,” “female” and “diverse.” The second question addresses age, offering seven options (1 ≤ 20, 2 = 20–30, 3 = 31–40, 4 = 41–50, 5 = 51–60, 6 = 61–70 and 7 = 71–80). The third question concerns marital status, with three response options (1 = single; 2 = married; 3 = living in a partnership). The next question refers to the elected roles, with five options (1 = lead-link, 2 = rep-link, 3 = facilitator, 4 = secretary, 5 = none of the above). The final question queries the industry of the employee.

The Bern Illegitimate Tasks Scale (BITS; Semmer et al., 2010) assesses illegitimate tasks. This study used two items that were proposed as a short measure by the author(s) of the BITS: one of them measures unnecessary and unreasonable tasks. For unnecessary tasks, participants were asked, “Do you have work tasks to take care of, which keep you wondering if they have to be done at all?” For unreasonable tasks, participants were asked, “Do you have work tasks to take care of, which you believe should be done by someone else?”

*Appreciation* was measured by asking the participants to assess to what degree they agreed with the following statement: “I generally feel appreciated at my workplace.” This item also used a 7-point Likert scale (Jacobshagen et al., 2008).

The short version of the MRS inventory by Schallberger and Venetz (1999) was used in this study to measure the Big Five personality dimensions. The short version of the MRS inventory is based on the MRS inventory by Ostendorf (1990) and Ostendorf and Angleitner (1992). The short version of the MRS inventory consists of 30 bipolar items with a 6-point Likert scale. Cronbach’s alpha for all five personality scales ranged between α = 0.65 and α = 0.81.

To measure the *person–organisation fit,* the four items were based on the scale developed by Saks and Ashforth (2002), using a 5-point Likert scale ranging from 1 (to a very little extent) to 5 (to a very large extent). This section included questions such as “To what extent are the values of the organisation similar to your own values?” Cronbach’s alpha for all four items was α = 0.89.

Additionally, one item to measure *satisfaction with holacracy* was included. *Holacracy satisfaction* was measured by asking participants the following: “How satisfied are you with holacracy as a form of organisation?” The response scale was a 5-point Likert scale from 1 (very satisfied) to 5 (very unsatisfied).

The questionnaire was designed with the programme Socisurvey.^1^ The study was conducted online and consisted of three different phases. The first segment contained demographic questions, and the second part consisted of inquiries regarding personality. More precisely, the survey recorded the Big Five. The participants answered questions about their satisfaction with holacracy and person –organisation fit. The survey lasted approximately 7 mins per person. The online survey consisted of a pool of 38 questions, and the survey period was between 12/20/2020 and 1/15/2021. Initially, online research was conducted to identify companies that publicly stated they were working with *holacracy*. Participants received the survey link through LinkedIn by contacting employees who stated they work at a *holacratic* company or through the human resources departments. All participants were informed that the prerequisite for participation was employment at a holacratic company.

The questionnaire began as soon as a participant clicked on the link. They first saw a welcome text informing them about the purpose of the study, a brief overview of the questions and the anonymity and voluntariness of the study. They responded to demographic questions at the beginning, followed by an information text that prepared the participants for the personal questions. They then responded to inquiries related to the Big Five. Participants answered all five dimensions on one page, then responded to questions on *holacracy satisfaction*, *person–organisation fit* and all remaining questions together on one page. The average time needed to answer the survey was 7 mins 30 s (*M* = 441.53, *SD* = 112.79, unity = seconds).

### 2.3. Data analysis procedures

#### 2.3.1. Propensity-matching approach

To investigate whether the work conditions differ between holacratic and traditional organisations, this study used the propensity-matching approach to compare the findings from the original study. Some data were analysed from a Swiss study called *Job-Stress-Index 2020.* Since 2014, Health Promotion Switzerland has been regularly collecting key figures on work-related stress and its correlation with the health and productivity of employees in Switzerland. The study was conducted in February 2020.

The total number of participants was 2,846 (N = 2,846), of whom 1,389 were female and 1,457 male. The propensity-matching approach involves finding a twin for each participant so that the results can be compared more efficiently. This proposal has its basis on three categorical variables: age, gender and industry. Since the two studies differed slightly in their response options for the item industry, a few response options were combined by the JSI into one industry. As an example, in the present study there was the industry “Finance, Real Estate and Insurance” as an answer option. In the JSI data set, there was the industry of “8. Credit and Insurance” and “9. Real Estate, other economic services.” These two industries were then combined into one. By using this principle, it was possible to find a twin for 52 of a total of 115 participants, who gave identical answers in all three categories. This allowed for a T-test for dependent samples to be conducted to compare both groups.

Regarding the data analysis, this study used R Studio version 1.2.5001. In the first step, answers from 20 participants were removed due to incompleteness. Moreover, irrelevant variables, such as the starting time of each participant, were removed. Building the scales first involved recoding negatively keyed items. Following this, each scale was created by summarising the relevant items. An alpha level of 0.05 was used, and the tests were two-tailed.

## 3. Results

### 3.1. Descriptive statistics

In the first step, we analyzed whether typical control variables like age and gender are correlated to the main concept’s person-organisation fit, holacarcy satisfaction and illegitimate tasks. No gender differences could be found regarding the main concepts. For age, only person-organisation fit showed a significant positive correlation [*r* (94) = 0.2, *p* = 0.49; Tables 1, 2].

**Table 1:** Overview of all research hypotheses including a separation in the two groups comparison between holacarcy organisations and traditional organisations and analysis only within holacracy organisations.

**Comparison between Holacracy-organisations & traditional organisations**	**Analysis only within Holacracy-organisation**	
**Hypothesis 1:** fewer illegitimate tasks inHolacracy organisations +	**Hypothesis 3:** positive correlation betweenextraversion and PoF -	
**Hypothesis 2:** higher appreciation in Holacracy organisations +	**Hypothesis 4:** negative correlation between neuroticism and PoF +	
	**Hypothesis 5:** positive correlation between openness to experience and PoF -	
	**Hypothesis 6:** positive correlation between conscientiousness and PoF -	
	**Hypothesis 7a:** positive correlation between agreeableness and PoF (+)	**Hypothesis 7b:** positive correlation between agreeableness and Holacracy Satisfaction +

Hypotheses marked with a “+” indicate that results supported the hypothesis, Hypotheses marked with a “-” indicate that results rejected the hypothesis, Hypotheses marked with a “+” indicate that results were marginally significant (*p* = 0.06).

**Table 2:** Descriptive statistics and reliability of the scales.

Variable	*M*	*SD*	Min	Max	Cronbachs α
Openness	4.51	0.63	3.17	6	0.73
Extraversion	4.17	0.87	2	6	0.81
Neurotizism	2.43	0.62	1.17	4	0.67
Conscientiousness	4.64	0.68	2.5	6	0.79
Agreeableness	4.63	0.58	2.83	5.67	0.65
Person-organisation-fit	3.94	0.72	1.5	5	0.89
Illegitimate tasks	2.45	0.81	1	5	
Appreciation	5.36	1.36	1	7	

*N* = 95, M = Mean, SD = Standard Deviations.

### 3.2. Hypothesis testing

Hypotheses 1 and 2 investigated the differences between holacratic and traditional organisations and therefore involve a comparison of employees in holacratic vs. traditional organisations; hypotheses 3 to 6 focused on the predictors for person–organisation fit, and used only the sample of people working in holacratic organisations (Table 3).

**Table 3:** Pearson correlations of person–organisation fit and holacracy satisfaction with personality traits.

Variable	Person-organisation-fit	Holacracy satisfaction
Extraversion	0.01	−0.01
Neurotizism	−0.27**	−0.32**
Conscientiousness	0.05	0.11
Agreeableness	0.19	0.23*
Openness	0.16	0.08
Unreasonable tasks	−0.21*	−0.13
Unnecessary tasks	0.32**	−0.26*
Person-organisation-fit	1	0.44**
Appreciation	0.55**	0.24*

*N* = 95, *p < 0.05, **p < 0.01.

#### 3.2.1. Hypothesis 1

**Figure 1:**
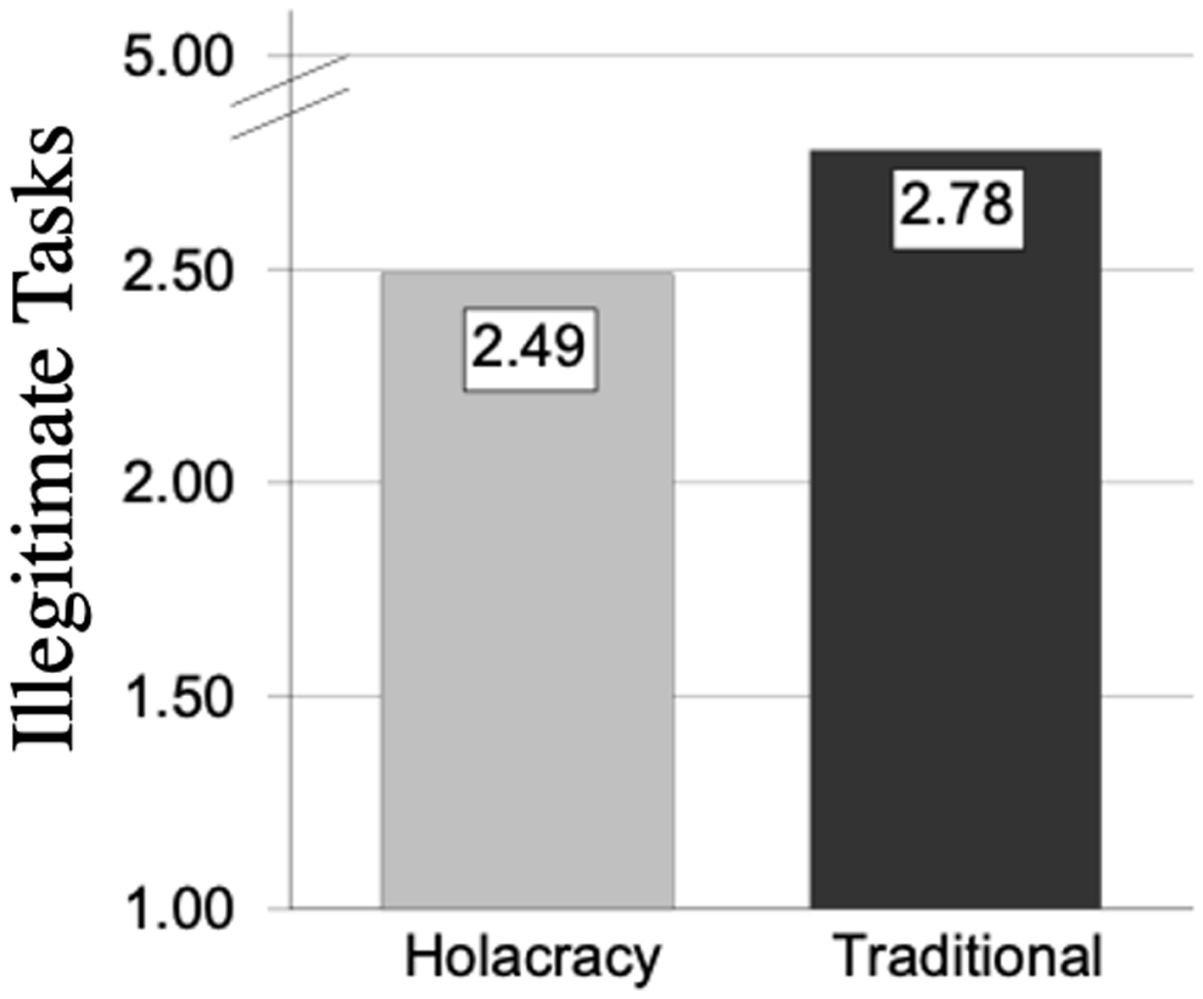
Main values of illegitimate tasks in holacratic organisations and traditional organisations.

To investigate whether people who work in holacratic companies significantly experienced fewer illegitimate tasks than people who worked in traditional companies, a *T*-test was performed to check significant group differences. The results revealed significant differences between both groups t (53) = −2.04, *p* < 0.05, *d* = 0.57, in which employees working in holacratic organisations showed lower mean values (2.49, SD = 0.65) than employees working in traditional organisations (2.78, SD = 0.64; Figure 1).

#### 3.2.2. Hypothesis 2

**Figure 2:**
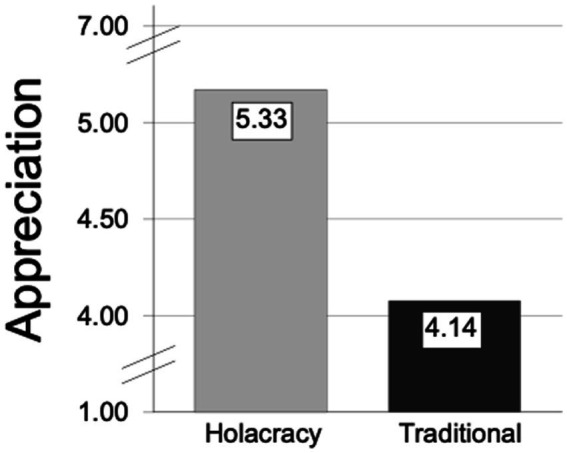
Main values of appreciation in holacratic organisations and traditional organisations.

It was assumed that people who worked in holacratic companies would experience significantly higher appreciation than people who worked in traditional companies. A T-test was conducted to verify significant group differences between holacratic organisations and traditional companies. The results revealed significant group differences t (53) = 4.86, *p* < 0.001, *d* = 0.81, in which employees working in holacratic organisations showed a higher mean level (5.33, SD = 1.41) than employees working in traditional organisations (4.14, SD = 0.81; Figure 2).

#### 3.2.3. Hypothesis 3

This hypothesis investigated the correlation between *person–organisation fit* and *extraversion.* A Shapiro–Wilk test was conducted to check the normality in the distribution of the data. Due to the normal distribution for both variables (*p* > 0.05), a Pearson correlation was performed to test the first hypothesis. The results did not show a significant correlation between *extraversion* and *person–organisation fit* [*r* (94) = 0.01, *p* = 0.95].

#### 3.2.4. Hypothesis 4

This hypothesis investigated the correlation between *neuroticism* and *person–organisation fit*. A Shapiro–Wilk test was conducted to check the normality in the distribution of the data. Due to the normal distribution for both variables (*p* > 0.05), a Pearson correlation was conducted to test the first hypothesis. The results showed a significant correlation between *neuroticism* and *person–organisation fit* [*r* (94) = −0.27, *p* < 0.001]. The results of the linear regression showed that *neuroticism* could be identified as a significant predictor for *person–organisation fit* [b = −4.72, t (93) = −0.32, *p* = 0.006. *R^2^* = 0.07 *F* (1, 93) = 3.6, *p* = 0.006; Table 4].

**Table 4:** Regression analysis summary for neuroticism predicting person–organisation fit.

Variable	B	ß	t	*p*	R^2^	Adjusted R^2^	F Statistic
Overall model					0.07	0.06	7.77 (df = 1;93)
(Constant)	4.77		16.28	*p* < 0.01			
Neuroticism	−0.32	−0.27	−2.78	0.006			

#### 3.2.5. Hypothesis 5

The fifth hypothesis investigated the correlation between *openness to experience* and *person–organisation fit*. Again, a Pearson correlation was conducted due to normality in the distribution of the data (Shapiro–Wilk test *p* > 0.05). There was no significant correlation found between *openness to experience* and *person–organisation fit* [*r* (94) = 0.16, *p* = 0.11].

#### 3.2.6. Hypothesis 6

The sixth hypothesis investigated the correlation between *conscientiousness* and *person–organisation fit*. A Pearson correlation was conducted due to normality in the distribution of the data (Shapiro–Wilk test *p* > 0.05). The results showed no significant correlation between *conscientiousness* and *person–organisation fit* [*r* (94) = 0.05, *p* = 0.62].

Hypotheses 7a and 7b analysed the relationships between *agreeableness* and *person–organisation fit* and between *agreeableness* and *satisfaction with holacracy.*

#### 3.2.7. Hypothesis 7a

A Pearson correlation was conducted to analyse the correlation between *agreeableness* and *person–organisation fit.* The results showed marginal significant positive correlation between *agreeableness* and *person-organisation fit* [*r* (94) = 0.19, *p* = 0.06]. A linear regression was conducted to investigate whether *agreeableness* is also a predictor for *person–organisation fit*. The relationship was visualised to provide a first impression regarding these variables. The linear relationship was rather weak. The results of the linear regression showed that *agreeableness* could not be identified as a significant predictor for *person–organisation fit* [b = 2.83, t (93) = 0.23, *p* = 0.06. *R^2^* = 0.04 *F* (1, 93) = 3.6, *p* = 0.06] when holding tight to the significance level of 0.05 (Table 5).

**Table 5:** Regression analysis summary for agreeableness predicting holacracy satisfaction.

Variable	B	ß	t	*p*	R^2^	Adjusted R^2^	F Statistic
Overall model					0.05	0.04	4.61 (df = 1;93)
(Constant)	2.12		2.82	*p* < 0.01			
Neuroticism	0.35	0.21	2.14	0.034			

#### 3.2.8. Hypothesis 7b

In addition, for this hypothesis, a Pearson correlation was conducted to investigate the correlation between *agreeableness* and *satisfaction with holacracy*. The results showed a significant positive correlation between *agreeableness* and *satisfaction with holacracy* (*r_s_* (94) = 0.22, *p* = 0.03). The linear relationship—weak to moderate—between these two variables was plotted for further analysis.

Following this, a linear regression was performed. The predictor was *agreeableness*, and the dependent variable was *satisfaction with holacracy*. *Agreeableness* was shown to be a significant predictor for *satisfaction with holacracy* [b = 2.13, t (93) = 0.34, *p* = 0.03. *R^2^* = 0.05 *F* (1, 93) = 4.61, *p* = 0.03; Table 6].

**Table 6:** Regression analysis summary for agreeableness predicting person-organization fit.

Variable	B	ß	t	*p*	R^2^	Adjusted R^2^	F Statistic
Overall model					0.04	0.03	3.58 (df = 1;93)
(Constant)	2.83		4.79	*p* < 0.01			
Neuroticism	0.24	0.19	1.89	0.061			

The normal distribution hypothesis of the residuals was analysed, which is an important factor for the validity of the tests. The test showed that the normal distribution hypothesis seemed to be valid in this case.

## 4. Discussion

Holacracy denotes a modern form of organisational governance. The first part of this study focused on differences in work conditions between holacratic and traditional organisations. The goal was to understand better these new forms of organising companies by comparing important work conditions. The second part of the study focused on different personality traits as predictors for person–organisation fit and satisfaction with holacracy. The objective was to identify relevant personality traits that predict satisfaction and individual fit with holacracy.

The goal of hypotheses 1 and 2 was to determine whether there are systematic differences in illegitimate tasks and appreciation between holacratic and non-holacratic companies. For this study, the task stressor illegitimate tasks and resource appreciation were used. Illegitimate tasks are those that an employee evaluates as either unnecessary or unreasonable, i.e., they feel that the task assigned does not fit their role (Semmer et al., 2015).

The first hypothesis assumed that employees working in holacratic companies would experience fewer illegitimate tasks on average than employees working in traditional companies because employees can determine their own tasks due to the lack of hierarchies and dynamic processes. A significant difference was found, with employees in holacratic companies having fewer illegitimate tasks on average than employees in non-holacratic companies. These results were expected and confirmed the assumption that employees in holacratic companies rarely complete tasks that do not fit their role due to their high level of personal responsibility. This finding is very interesting, as studies have shown that if employees must perform illegitimate tasks too often, this can lead to stress, dissatisfaction and, in the worst case, termination (Semmer et al., 2015). Companies should therefore ensure that employees can either choose their tasks independently or that their supervisors know properly the role profiles of their employees, assign them tasks that fit their role and explain when illegitimate tasks are unavoidable. In addition to that, companies should ensure that supervisors understand the roles of their employees and, consequently, reduce illegitimate tasks (Semmer et al., 2010). Another explanation for why employees in holacratic companies experience fewer illegitimate tasks could result from a study from Björk et al. (2013). They showed that the more the organisation was characterised by competition for resources between units, unfair and arbitrary resource allocation and an obscure decisional structure, the higher the illegitimate tasks score of the participants was. With holacracy, power is distributed throughout a concrete organisational structure, giving individuals and teams freedom while staying aligned with the organisation’s purposes. The organisation in circles could lead to an equal and fair distribution of resources, and decision-making processes should be made transparent (Robertson, 2015).

The second hypothesis examined whether employees in holacratic companies experienced higher appreciation than employees in traditional companies. The results showed that there was a significant difference between the two groups, with the holacratic group showing higher average values on the appreciation scale. This confirms the second hypothesis. One explanation for this could be that good cooperation and respectful interaction between colleagues in holacratic companies has a high priority and that the lack of hierarchy tends to create a sense of community. Everyone contributes to the success of the company and does their best to support their team. In addition to lower illegitimate tasks that function as stressors, there is also a higher level of appreciation that functions as a resource. Appreciation was shown to buffer the link between illegitimate tasks and well-being (Stocker et al., 2010). Supervisors often assign illegitimate tasks and are criticised for expressing sparse appreciation to followers (Kottwitz et al., 2019). Future studies should locate the sources of illegitimate tasks and appreciation in holacracy because there are fewer or no supervisors (Robertson, 2007). The results of the first and second hypotheses indicate that in holacarcy companies, working conditions are to the advantage of employees. Higher perceived appreciation and less illegitimate tasks could therefore have a positive impact on employees’ performance and thereby on the economic success of the company. This is assumption is supported by a just recently published study, that analyzed the performance of democratically structured enterprises and found out that out of 83 investigated enterprises 50 showed no signs of degeneration or even degeneration tendencies (Unterrainer et al., 2022). Better working conditions and thereby a better performance of the employees may be a potential explanation for this. The fact that the working conditions in holocratical companies seem to be better than in traditional companies is also interesting, because in the past the attempt to hand over power to the employees has already failed. The former Yugoslavia can be mentioned here as an example. Here, the introduction of self-management had rather negative effects, as there was a clash of interests between the organisation and the employees and, in some cases, exploitation of the employees (Koyama, 2013).

The third hypothesis investigated whether extraversion is a predictor for person–organisation fit. This assumption was made because holacracy requires all employees to interact a lot with other colleagues and to defend their positions, for example when denying a certain task due to a lack of existing hierarchies. Employees who show a typical personality trait for high extraversion should feel more comfortable in such an environment and experience a higher fit. The results, however, did not confirm this hypothesis, as no correlation between extraversion and person–organisation fit was found. The results, therefore, do not align with hypothesis three. One explanation is that only working in a holacratic company does not necessarily mean that self-confidence is required, and hence extraversion has no impact on the person–organisation fit. It is probable that employees who have roles mainly consisting of tasks that do not require exchanges with colleagues and whose roles are clear, such as programming, do not need to have high values for extraversion. This means that even with low extraversion employees can still experience high person–organisation fit.

Moreover, the results of a meta-analysis that investigated differences between managers and entrepreneurs using the Five Factor Model showed that results regarding extraversion across the studies were characterised by variability. Zhao and Seibert (2006) found relevant differences for all dimensions except extraversion, as the CI was too wide, and the CRI indicated mixed results across the studies that were part of the meta-analysis. The role of extraversion in a holacratic environment remains open for the time being.

The fourth hypothesis investigated the role of neuroticism regarding person–organisation fit. Neuroticism emerged as the strongest correlation for person–organisation fit. Moreover, the results of the linear regression confirmed the assumption that neuroticism is also a significant predictor for person–organisation fit. The results were expected and are aligned with other studies that investigated the role of neuroticism in the work context: in their meta-analytic review, Judge et al. (2002) investigated the role of the Big Five and job satisfaction. They also found neuroticism to be the strongest correlate for job satisfaction. These results can be explained by the fact that people who show higher values for this dimension are likely to experience more negative life events, including at work. This will again influence their satisfaction and their experienced fit. In addition, high values for neuroticism mean that someone is often anxious and hostile. Working in a company where everything is about teamwork and interaction is thus not a good fit for such a person.

Whether openness to experience correlates with the experienced fit was the goal of the fourth hypothesis. The results did not show any relationship between those two variables and thus contradict the assumption of the hypothesis. The positive relationship was expected because people who have high values for this dimension typically are creative and prefer political liberalism. Due to the lack of hierarchy, holacracy allows employees to fully use their creativity and develop their own ideas. Moreover, the approach is closely related to political liberalism, so people who believe in this political approach were expected to experience a high person–organisation fit. A possible explanation for the result could be that when working with holacracy, the practice is not as creative and liberal as expected. It should be kept in mind that there are still many rules that must be followed, and through some special roles like the “lead-link” or “the rep-link,” a certain kind of hierarchy still exists. Also, through the constitution, the general procedure is strictly organised and could prevent creativity and the feeling of liberalism for some employees. Apart from that, there are still positions for which no creativity is needed.

The sixth hypothesis investigated the correlation between conscientiousness and person–organisation fit. No significant relationship was found. The results were not expected and are not in line with the hypothesis. In holacratic companies, self-organisation is expected to play one of the most important roles, as it enables a person to handle better the loose structures and high level of responsibility. If someone has high values for this personality dimension, they may handle holacracy better and feel that the company matches their own competences. But, again, this was not the case for this study. The results indicate that self-organisation is not a necessary skill for an employee who is working in a holacratic company. A possible explanation for this result is that holacracy still involves many requirements, such as a high number of meetings, despite the lack of hierarchies (Bernstein et al., 2016). This means that a high level of self-organisation is not necessarily a prerequisite for holacracy, and employees will not necessarily struggle if they have poor organisational skills. This contradicts the statement of Van De Kamp (2014), who assumed that self-organisation is important to deal with holacracy.

One of the five dimensions—agreeableness—was of particular interest in this study. This dimension could play a meaningful role in holacratic companies. A study by Seibert and Kraimer (2001) found that people with high scores for this dimension tend to earn less and are more likely to be less satisfied with their careers than people with low scores. This can be explained by the fact that people with high scores on this scale need harmony and are more committed to others than to themselves. In traditional companies, this behaviour would probably do them more harm than good in the long term. In holacratic companies, it is not about the individual career because without titles and hierarchies, the career in the company plays a subordinate role. Rather, teamwork and mutual consideration are more relevant and lead to recognition and respect. Selfishness or selfish behavior is likely to be given much less space than in more traditional approaches. As previously described, Templer (2012) found in his study that agreeableness also plays a different role in Eastern than in Western European cultures. The same principle applies: if a person wants to pursue a career in Europe or the United States, they should not pay too much attention to their colleagues to gain recognition. The same behaviour would not lead to recognition in Asian cultures; in contrast, the common good comes first. Someone who puts community first will find recognition and respect.

Hypotheses 7a and 7b analysed the relationships between agreeableness and person–organisation fit and between satisfaction and holacracy. Hypothesis 7a focused on agreeableness and person–organisation fit. The results showed a significant positive correlation between these two variables and are aligned with the hypothesis. The linear regression showed a marginally significant result, as the value of p was 0.01 above the suggested 0.05 value. There is a lot of criticism of this rigid limit (Amrhein et al., 2017).

Hypothesis 7b investigated the relationship between agreeableness and satisfaction with holacracy. The results confirmed the findings from hypothesis 7a, as they also showed a positive, significant relationship between the two variables. The results of the linear regression confirmed the assumption that agreeableness is also a significant predictor for satisfaction. However, the explained variance was rather small.

These results are in line with those from a study from Templer (2012), which investigated whether agreeableness plays a more important role in job satisfaction in collectivist societies compared with individualist societies. The results suggested that in collectivist societies, agreeableness plays a prominent role in the explanation of job satisfaction. Templer (2012) assumed that in collectivistic societies agreeable individuals are rewarded for having harmonious relationships at work and not being involved in conflicts. In individualistic societies, a low level of tolerance is a sign of strength and can lead to a fight for a higher position, which leads to reassurance and rewards. Companies that work with holacracy may be more like a collectivist society, because in both contexts making good impressions and displaying prosocial behaviors to in-group members is very important. Being agreeable and helpful is therefore an important prerequisite. In contrast to that, individuals living in western societies and working in traditional hierarchical structures might also be rewarded and get ahead of others for being disagreeable and entering into conflict with others if they are perceived as performers by their superiors (Templer, 2012). In addition, employees might feel that the structures within holacracy fit their personality well, resulting in a high value for the person–organisation fit scale. These results indicate that agreeableness could be a beneficial personality trait for employees working in holacratic companies to experience a high person–organisation fit.

### 4.1. Theoretical implications

This study raises the question of the extent to which findings from earlier studies, where traditional hierarchies were still normal, can be applied to new concepts such as holacracy. When the CEO of Zappos announced that they would switch to holacracy, he emphasised that all employees could now behave like entrepreneurs. The entrepreneurship literature states that entrepreneurs are often characterised by high values for conscientiousness and openness, and low values for agreeableness and neuroticism (Zhao and Seibert, 2006). This contrasts with the results of this study, which associated high values on the compatibility scale with a high level of person–organisation fit. The feasibility of the Big Five in different contexts should be considered when determining the design of future studies. Some studies highlight the relevance of cultural context (Triandis and Suh, 2002; Templer, 2012). The results of the present study indicate that even if the cultural context remains the same, other circumstances related to a new form of company organisation can change the impact of the Big Five.

In addition, the relevance of illegitimate tasks and appreciation becomes clear. The two constructs seem closely linked and play a particular and important role, especially for the younger generations, Y and Z. Studies have found that the relevance of hard factors such as salary and career opportunities decrease, and factors such as the implementation and recognition of own ideas and appreciation become more important (Deutschland, 2015). The question arises as to whether appreciation from superiors or colleagues is more important. A study from 2015 found that the appreciation of colleagues is particularly important for Generation Z. This may explain why appreciation values in holacratic companies are higher than in traditional ones. Researchers should consequently consider that when general appreciation is examined, who the appreciation is coming from is clearly defined. In addition, researchers should try to find out which circumstances and criteria lead to employees in holacratic companies feeling more valued and less likely to carry out illegitimate tasks since both constructs have an enormous influence on employee satisfaction and on the success of the company (Semmer et al., 2010, 2015; Eatough et al., 2016; Kottwitz et al., 2019).

### 4.2. Practical implications

Based on the results of this study, human resources managers should conduct personality tests that investigate the Big Five because results suggest that agreeableness and neuroticism predict person–organisation fit, which influences job satisfaction and the intention to quit (Kristof-Brown et al., 2005). Other personality traits, such as extraversion, openness to experience or conscientiousness showed no correlation with person–organisation fit, which means that they probably do not play a general role in employee satisfaction with holacracy.

Interesting results from this study emerged from hypotheses 7a and 7b, which highlight the relevance of the variable agreeableness in the context of holacracy. Human resources managers should therefore watch for applicants who do not show a behaviour that indicates a high potential for conflict readiness. Moreover, neuroticism also significantly correlated negatively with the dependent variable person-organisation fit.

In addition, when comparing holacratic and traditional companies, it was shown that employees in a holacratic organisation rarely must do illegitimate tasks and generally feel more valued at their workplace than employees in traditional companies. These results suggest that approaches like holacracy are not getting a lot of attention without reason. Self-organisation, instead of strict guidelines and strict hierarchies, seems to meet the needs of the younger generations (Lang and Scherber, 2018). Traditional companies should therefore question their existing structures and change their processes in such a way that employees can choose their roles by themselves. In addition, flat hierarchies seem to lead to colleagues treating each other with attention and respect despite a lack of appreciation from superiors. Companies should therefore take care to create an atmosphere and a culture in which colleagues respect and value one another. This result fits with the goal of holacracy: to create a sense of community, which leads to high productivity and agility in order to offer successful products and services.

### 4.3. Limitations and future research

The following limitations must be considered. First, a main limitation was the cross-sectional data. Changes in the perceived person–organisation fit may occur over time and differ from day to day. So, future studies should include more than one measurement point. Second, the participants were mainly recruited *via* LinkedIn, meaning that people without a LinkedIn account were excluded from participation. In their study from 2014, McPeake et al. (2014) argued that one disadvantage of online surveys is that it is not suitable for everyone in the same way and thereby excludes participants, a bias which is also known as selection bias. In addition, the findings can only be applied to Germany, Austria and Switzerland. It should also be noted that employees are often requested to participate in online surveys, especially when working in a company that uses a highly modern approach. This assumption is supported by experiences from this study, as many participants refused to participate with the explanation that they were too often invited to do so. Future studies might then seek to invite participants to a laboratory and offer compensation to increase their motivation. Future studies should also note how long an employee has already worked with holacracy, as valid results may not be produced over a short period. In addition, researchers should further address which factors explain some people being satisfied with holacracy and having a high person–organisation fit as compared with others. Out of five dimensions, only two showed correlations with the dependent variables. It is of great interest to determine which other factors predict person–organisation fit.

For example, more specific personality traits, such as perseverance, creativity and adaptability, are believed to be predictors for entrepreneurship (Fueglistaller et al., 2019) and could therefore influence the perceived person-organisation fit. In addition, external factors, such as the practical implementation of holacracy, should be examined as influencing factors for employee satisfaction. For example, a start-up called Blinkist reports that they are using a slightly modified form of holacracy (Klein, 2015). This might lead to a bias in the results. As mentioned above, the results indicate that even if the cultural context remains the same, other circumstances related to a new form of company organisation can change the impact of the Big Five. Therefore, future studies should carefully consider the context in which the study is being conducted.

As a last limitation, it should be mentioned that the results of hypotheses 1 and 2, which examined the differences between holacratic and traditional companies, could be explained by more room to manoeuver. However, holacracy is still partly structured by the constitution and other further rules. Future studies should include room to maneuver as a control variable.

## 5. Conclusion

The results of this study indicate that working conditions systematically differ between holacratic and traditional companies. Employees in holacratic companies experienced higher appreciation and less illegitimate tasks than workers in traditional organisations.

Moreover, an interesting finding is that high agreeableness showed significant results for all of the two dependent variables. The reason for this might be that companies that use holacracy foreground a feeling of community instead of individual well-being. As low agreeableness might otherwise lead to high person–organisation fit in individualistic societies, the holacratic approach is potentially similar to work environments in collectivist societies. The role that extraversion, openness to experience and conscientiousness were expected to play regarding their correlation with person–organisation fit were not confirmed and should be investigated in more detail in further studies.

## Data availability statement

The raw data supporting the conclusions of this article will be made available by the authors, without undue reservation.

## Ethics statement

The studies involving human participants were reviewed and approved by Ethics Committee of the University of Bern, Switzerland (Ethics no. 2019–01-00005). The patients/participants provided their written informed consent to participate in this study.

## Author contributions

LW, SG, and AE designed the study. AE structured the ideas. LW did the analyses. All authors contributed to the article and approved the submitted version.

## Conflict of interest

The authors declare that the research was conducted in the absence of any commercial or financial relationships that could be construed as a potential conflict of interest.

## Publisher’s note

All claims expressed in this article are solely those of the authors and do not necessarily represent those of their affiliated organizations, or those of the publisher, the editors and the reviewers. Any product that may be evaluated in this article, or claim that may be made by its manufacturer, is not guaranteed or endorsed by the publisher.

## Footnotes

^1^https://www.soscisurvey.de/
